# CellRemorph: A Toolkit for Transforming, Selecting, and Slicing 3D Cell Structures on the Road to Morphologically Detailed Astrocyte Simulations

**DOI:** 10.1007/s12021-023-09627-5

**Published:** 2023-05-03

**Authors:** Laura Keto, Tiina Manninen

**Affiliations:** Faculty of Medicine and Health Technology, Tampere University, Tampere, Finland

**Keywords:** 3D cell morphology, Astrocyte, Transform, Mesh, Point cloud, Computational tool

## Abstract

Understanding functions of astrocytes can be greatly enhanced by building and simulating computational models that capture their morphological details. Novel computational tools enable utilization of existing morphological data of astrocytes and building models that have appropriate level of details for specific simulation purposes. In addition to analyzing existing computational tools for constructing, transforming, and assessing astrocyte morphologies, we present here the CellRemorph toolkit implemented as an add-on for Blender, a 3D modeling platform increasingly recognized for its utility for manipulating 3D biological data. To our knowledge, CellRemorph is the first toolkit for transforming astrocyte morphologies from polygonal surface meshes into adjustable surface point clouds and vice versa, precisely selecting nanoprocesses, and slicing morphologies into segments with equal surface areas or volumes. CellRemorph is an open-source toolkit under the GNU General Public License and easily accessible via an intuitive graphical user interface. CellRemorph will be a valuable addition to other Blender add-ons, providing novel functionality that facilitates the creation of realistic astrocyte morphologies for different types of morphologically detailed simulations elucidating the role of astrocytes both in health and disease.

**Supplementary Information:**

The online version contains supplementary material available at 10.1007/s12021-023-09627-5.

## Introduction

Astrocytes are elaborate star-like cells embedded in a three-dimensional extracellular matrix with other glial cells, neuronal cells, and vasculature and play important roles in the brain from basic maintenance to higher information processing (Verkhratsky & Nedergaard, 2018). Computationally probing the functionality of astrocytes requires distinct approaches in comparison to those developed for neuronal cells. Astrocytes differ from neurons both in function and morphology: they do not fire action potentials and are characterized by a complex surface of nanoscopic processes (Verkhratsky & Nedergaard, 2018). Astrocytes have specialized processes that envelop neuronal synapses (Bushong et al., 2002), endfeet that connect them to vasculature (Takano et al., 2006), and differential spatial organization of intracellular components (Calì et al., 2019). Like neurons, whose branching structure influences the biophysical properties of neuronal networks (Cuntz et al., 2007), astrocytes have unique branching geometries that differ from astrocyte subtype and brain region to other (Khakh & Sofroniew, 2015). Calcium waves propagate along the astrocyte morphology and are affected by the branching geometry (Semyanov, 2019). While in general signaling occurs on longer time scales in astrocytes than in neurons, rapid calcium signaling on similar timescales to neurons is observed in specialized processes and endfeet of astrocytes (Stobart et al., 2018).

Studying the complex functionality of astrocytes requires computational models that capture their morphological details and enable reaction–diffusion modeling. However, only a few such models exist for astrocytes (Manninen et al., 2018), whereas hundreds of multicompartmental whole-cell models exist for neurons that capture most of their functionally important morphological characteristics. While single-compartmental astrocyte models have elucidated and confirmed some aspects of astroglial physiology, many important astrocytic functions, such as calcium waves, cannot be understood in detail without considering the complex astrocyte morphology (Semyanov, 2019). Studying astrocytic mechanisms behind many of the important processes in the brain that keep it healthy would be facilitated by computational models that capture the functionally relevant morphological characteristics of whole astrocytes. In addition, detailed computational models of astrocytes enable testing hypotheses which are difficult to perform experimentally and pave the way for deciphering the link between astrocyte morphology and function.

Morphologically detailed computational models of astrocytes can be implemented utilizing the same approaches and tools as developed for neurons, but these tools do not accommodate the characteristics of astrocytes. Neuronal models are often represented as skeletal models consisting of a hierarchical tree of linear segments. While the main stem tree structure of astrocytes can be represented as such skeletal models and traced with light microscopy (LM), the nanoscopic geometry of astrocytes that is highly functionally relevant is ignored. On the other hand, building models that capture all the details are often infeasible for simulations. The ramified, dense 3D nanoscale architecture of astrocytes, that is below the diffraction limit of LM, can be captured with electron microscopy (EM) where a tissue is sliced, each slice imaged, and morphologies reconstructed from the images (Nahirney & Tremblay, 2021). The coordinates of the astrocyte perimeter in each image give rise to a surface point cloud that represents the morphology. Alternatively, the morphology can be reconstructed from EM images as a polygonal surface mesh, which can be further converted into a volumetric mesh format. Preparing these morphologies for different types of simulations is aided by computational tools for visualizing, manipulating, repairing, and assessing the morphologies. An increasing number of such tools have been developed on Blender (https://www.blender.org/), a free and open-source 3D animation software widely used by scientists.

From manipulating biomolecules in 3D space to building cellular networks, Blender has been utilized as a platform for a diverse set of computational tools for neuroscience. Blender by default includes an extensive set of functionalities for manipulating 3D objects and allows the creation of novel tools via Python-based interpreter. These Blender-based add-ons include functionalities such as importing digital representations of biological structures in different formats, analyzing and improving their 3D representations, transforming them into other formats, and creating visualizations and animations. Blender-based add-ons aimed towards producing realistic reconstructions of different parts of the nervous system include, for example, CellBlender (Kerr et al., 2008) and NeuroMorphoVis (Abdellah et al., 2018) for neuronal cells and VessMorphoVis (Abdellah et al., 2020) for brain vasculature. NeuroMorphoVis also includes utility for constructing synthetic astrocyte morphologies (Abdellah et al., 2021).

We present here a new Blender-based add-on, the CellRemorph toolkit, that provides functionality for transforming, selecting, and subdividing astrocyte morphologies in ways not available in any other previously implemented computational tool. The tools provided in the CellRemorph toolkit facilitate the steps needed to build morphologically detailed astrocyte models for different types of simulations. The first tool in CellRemorph toolkit enables selecting effectively and precisely nanoprocesses either from a polygonal surface mesh or surface point cloud formatted astrocyte morphology. The second tool enables transforming an astrocyte morphology from a polygonal surface mesh into a surface point cloud and vice versa. The third tool enables subdividing an astrocyte morphology into segments equal either in surface area or volume. The tools are easy and intuitive to use via the Blender graphical user interface. While the provided tools are aimed towards manipulating astrocyte morphologies, depending on the application they can be useful for processing other types of cell morphologies as well.

In this study, we discuss the challenges in morphologically detailed modeling of astrocytes and the existing solutions. We give an overview of general morphological properties of astrocytes and the requirements they pose for developing computational models that accurately capture all their functionally important morphological characteristics, and how modeling astrocytes is different in comparison to modeling neurons. We review the different formats astrocyte morphologies can be presented in, their utility, and convertibility into other formats. We analyze existing computational tools potentially applicable for constructing, transforming, and validating astrocyte morphologies, with a focus mainly on tools developed on the Blender software. We present our novel Blender-based add-on for manipulating astrocyte morphologies on the road to morphologically detailed simulations. Lastly, we present the discussion and conclusions.

## From Imaging Astrocyte Morphologies to Computational Modeling and Simulation

Identification of astrocytes and reconstructing them into computational formats suitable for different types of simulations is enabled by a diverse set of computational tools and methods. This section starts with an overview of the morphological characteristics of astrocytes and the existing computational tools and platforms that facilitate the construction of computational models, after which the steps leading into different types of computational morphologies are discussed. These steps include construction, transformation, validation, and subdivision. Lastly this section gives an overview of the simulation tools applicable for astrocytes.

### Morphological Characteristics of Astrocytes

While all parts of the neurovascular unit, including astrocytes, neurons, and vasculature, have hierarchical branching topologies, astrocytes are characterized by an intricate arborization of very fine nanoscopic processes and endfeet, both of which neurons and vasculature do not possess. These nanoscopic processes enwrap neuronal synapses (Hama et al., 2004; Ventura & Harris, 1999) and the endfeet interface vasculature (Mathiisen et al., 2010). In contrast to neurons, the surface-to-volume ratio in astrocytes is markedly higher (Calì et al., 2019). The surface area and volume of astrocytic segments are essential, since they affect the efficacy of astrocytic communication. The diffusion and localization of molecules are affected by the curvature and ultrastructure of astrocytes (Rangamani et al., 2013). Astrocyte morphologies feature microdomains consisting of thin necks and high surface areas that separate cellular events (Grosche et al., 1999). Astrocyte morphologies usually consist of several larger processes with secondary or tertiary branching that form a so-called stem tree of the astrocyte visible with LM (Khakh & Deneen, 2019). Up to thousands of smaller structures, called nanoscopic processes, are attached to the astrocytic stem tree (Khakh & Deneen, 2019). Since these nanoscopic processes are below the diffraction limit (0.3–0.5 µm) of LM, appearing as a cloudy structure, individual nanoprocesses can be resolved only with EM (Grosche et al., 1999). The shapes of astrocytic stem trees and their nanoscopic geometries vary greatly from brain region to another, accommodating different functional requirements (Khakh & Sofroniew, 2015). While in general the tissue domains of individual astrocytes do not overlap, astroglial cells from the cerebellum, for example, do overlap with each other (Grosche et al., 2002).

### Computational Tools

**Fig. 1:**
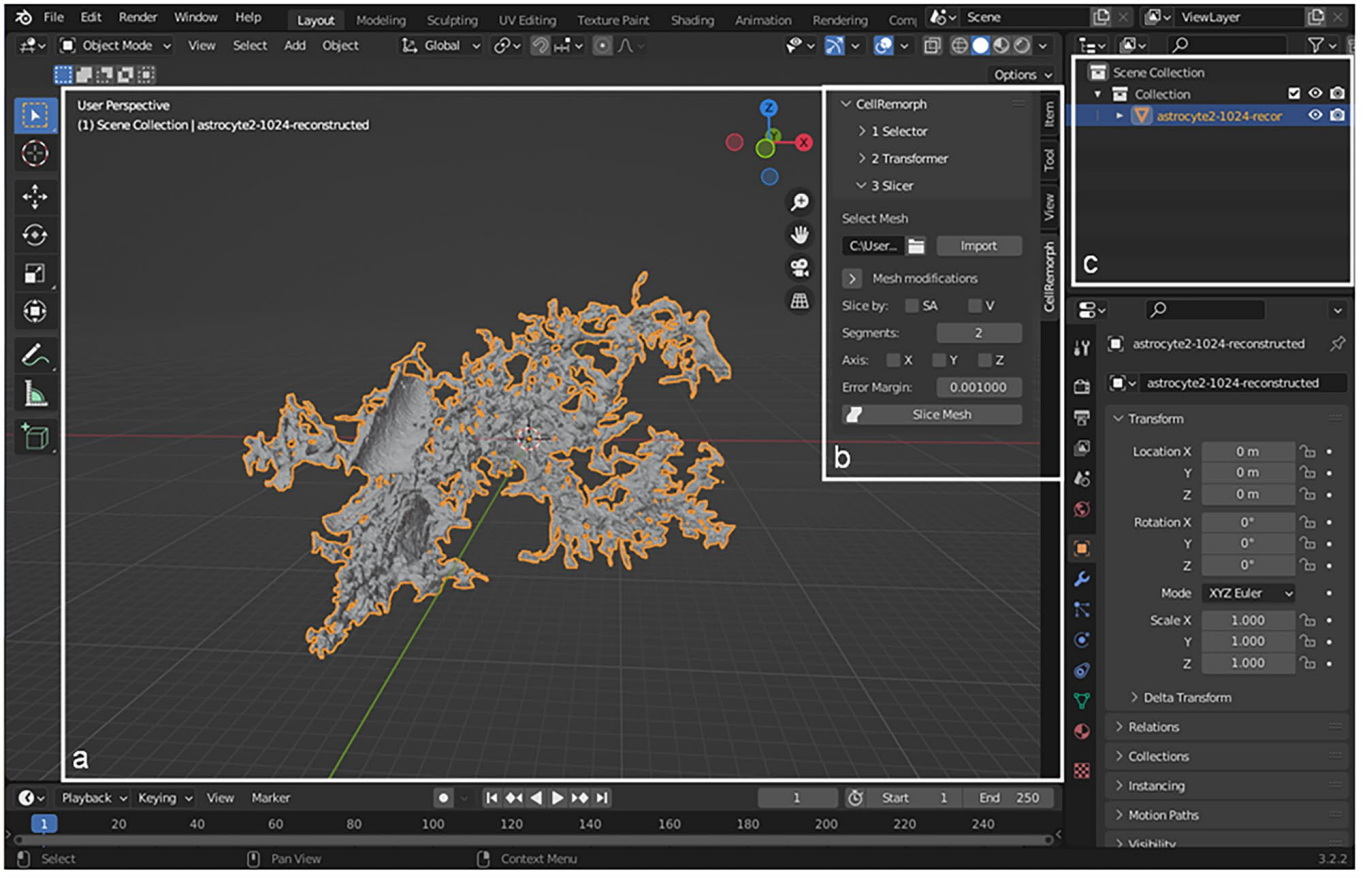
Blender user interface displaying an astrocyte morphology imported with the CellRemorph add-on. **a** Blender 3D viewport where the synthesized astrocyte polygonal surface mesh morphology obtained from the NGV Portal (https://bbp.epfl.ch/ngv-portal/anatomy/reconstruction-data/; Abdellah et al., 2021; Calì et al., 2019; Zisis et al., 2021) is displayed and can be rotated in 3D space. **b** The custom add-on, CellRemorph is displayed in the sidebar. **c** Scene collection contains all the objects present in the 3D viewport

Computational tools have been developed for all the different purposes from tracing cell morphologies from images to simulating the dynamical behavior of morphologically detailed cells. Professional 3D animation software platforms include, for example, Cinema 4D, Maya, and Blender. Since Blender is the only free open-source software platform of these three, tools developed on Blender are our main focus in this study, but we also list and give examples of other tools, both commercial and free open-source tools. Blender by default includes a 3D viewport useful for manipulating and examining cell morphologies in 3D space (Fig. 1a). Computational 3D analysis tools can be integrated into Blender as add-ons and displayed in the sidebar next to the 3D viewport (Fig. 1b). Cell morphologies imported into a specific Blender scene can be accessed from a scene collection where they are listed (Fig. 1c). Next, we present examples of the commonly used computational tools (Table 1) and Blender-based add-ons (Table 2) developed for different purposes.

**Table 1:** Examples of commonly used non-Blender computational tools for constructing and manipulating 3D cell morphologies. Different commercial and open-source tools are categorized based on their functionalities. EM and LM denote electron microscopy and light microscopy, respectively

**Stage**	**Functionality**	**Tool**
**Construction**	Tracing (EM)	Ilastik (Kreshuk et al., 2011)
		TrakEM2 (Cardona et al., 2012)
		webKnossos (Boergens et al., 2017)
	Tracing (LM)	FARSIGHT (Luisi et al., 2011)
		FilamentTracer^a^
		Neurolucida (Glaser & Glaser, 1990)
		Neuromantic (Myatt et al., 2012)
		Simple Neurite Tracer (Longair et al., 2011)
		TREES toolbox (Cuntz et al., 2010)
		Vaa3D (Peng et al., 2010, 2014)
	Synthetizing (skeletal morphologies)	NEURON CellBuilder (Carnevale & Hines, 2006)
		TREES toolbox
**Validation**	Visualizing	Abstractocyte (Mohammed et al., 2018)
		Brayns^b^
		NeuroAnatomy Toolbox (Bates et al., 2020)
		Open Source Brain (Gleeson et al., 2019)
		pyNeuroML^c^
		TREES toolbox
		Vaa3D
	Optimizing	Vaa3D
	Morphometrics	L-Measure (Scorcioni et al., 2008)
		NeuroAnatomy Toolbox
		TREES toolbox
		Vaa3D
**Transformation**	Skeletal to skeletal	Cvapp (Cannon et al., 1998)
		Cvapp for NeuroMorpho.org^d^
		hoc2swc^e^
		L-Measure
		neuroConstruct (Gleeson et al., 2007)
		NLMorphologyConverter^f^
		Open Source Brain
		pyNeuroML
	Skeletal to surface mesh	neuroConstruct
		Neurolucida
		Neuronize (Brito et al., 2013)
		NeuroTessMesh (Garcia-Cantero et al., 2017)
		SWC Mesher^g^
	Surface mesh to skeletal	VolRoverN (Edwards et al., 2014)
	Contour tracing to surface mesh	VolRoverN
	Surface mesh to volume mesh	VolRoverN
	Mesh complementary space to volume mesh	VolRoverN
**Subdivision**	Cutting volume or surface meshes	Vaa3D

^a^https://imaris.oxinst.com/products/imaris-for-neuroscientists

^b^https://github.com/BlueBrain/Brayns

^c^https://github.com/NeuroML/pyNeuroML

^d^https://github.com/pgleeson/Cvapp-NeuroMorpho.org/

^e^https://github.com/JustasB/hoc2swc

^f^http://neuronland.org/NL.html

^g^https://github.com/mcellteam/swc_mesher

**Table 2:** Blender-based tools for neuroscience. The tools are identified based on their functionalities. V and SA denote volume and surface area, respectively

**TOOL**	**BioBlender**^**a**^	**blenderNEURON**^**b**^	**BlendGAMer**^**c**^	**CellBlender**^**d**^	**cellPACK**^**e**^	**CellRemorph**^**f**^	**ePMV**^**g**^	**NeuroMorph**^**h**^	**NeuroMorphoVis**^**i**^	**NeuropilTools**^**j**^	**PAM**^**k**^	**Py3DN**^**l**^	**SWC2Blender**^**m**^	**VessMorphoVis**^**n**^
**Importation**														
Biomolecules	x				x		x							
Skeletal morphologies		x							x			x		x
Polygonal surface meshes			x	x		x		x						
Surface point clouds						x								
**Synthetization**														
Astrocytes									x					
Neuronal somata									x					
**Visualization**														
Illustration	x	x		x	x		x	x	x			x		x
Plotting				x										
Animation	x	x		x			x				x			
Image stacks with 3D mesh								x						
**Optimization**														
Detecting and repairing artifacts			x			x		x	x					x
Curvature estimation			x											
Mesh decimation			x			x								
Boundary marking			x											
**Morphometrics**														
Connectivity											x			
Spatial distribution												x		
Volume						x		x				x		
Surface area						x		x				x		
Lengths and distances								x	x		x			
**Transformation**														
Biomolecule to surface mesh			x											
Volume data to surface mesh							x							
Skeletal to surface mesh									x			x		x
Skeletal to Bezier curve													x	
Surface mesh to volume mesh									x					
Point cloud to surface mesh						x								
Surface mesh to point cloud						x								
**Subdivision**														
Free selection						x		x						
Subdivision by V/SA						x								
Subdivision of spines										x				
**Creation**														
Spiking neural networks											x			
Synapses		x												
Packing molecular structures					x									
**Platform**														
Blender	x	x	x	x	x	x	x	x	x	x	x	x	x	x
Other					x		x							

^a^http://www.bioblender.org/ (Andrei et al., 2012)

^b^https://blenderneuron.org/

^c^https://github.com/ctlee/gamer (Lee et al., 2020)

^d^https://github.com/mcellteam/cellblender (Kerr et al., 2008)

^e^https://www.autopack.org/ (Johnson et al., 2015)

^f^https://github.com/lauraketo/CellRemorph

^g^http://epmv.scripps.edu/ (Johnson et al., 2011)

^h^https://neuromorph.epfl.ch/ and https://github.com/neuromorph-epfl/neuromorph (Jorstad et al., 2015, 2018)

^i^https://github.com/BlueBrain/NeuroMorphoVis (Abdellah et al., 2018, 2021)

^j^https://github.com/mcellteam/neuropil_tools (Bartol et al., 2015)

^k^https://github.com/MartinPyka/Parametric-Anatomical-Modeling (Pyka et al., 2014)

^l^https://github.com/paulodecastroaguiar/py3DN (Aguiar et al., 2013)

^m^https://github.com/MartinPyka/SWC2Blender

^n^https://github.com/BlueBrain/VessMorphoVis (Abdellah et al., 2020)

### Construction

Constructing biological data into computational formats can be achieved either by reconstructing imaging data or synthesizing quantitative data. Reconstructing cellular data into computational formats usually consists of filling the cell with a fluorescent indicator, imaging the cell, tracing the neuronal or astrocytic processes, and saving the data into morphological format. The different methods for imaging and tracing, the variety of existing morphological formats, and the availability of astrocytic data in databases are discussed here. Alternative to reconstruction from images, synthesizing morphologies based on quantitative parameters, is also briefly overviewed.

#### Imaging

Imaging cells is the first step towards reconstructing 3D cell morphologies. Imaging methods are based either on LM or EM, and they range from manual to semi-automatic and automatic. Different levels of precision are acquired depending on the method used. LM methods for visualizing cells can be divided into two main categories: bright field microscopy and fluorescence microscopy. Common techniques for enhancing the visualization of cells are phase contrast imaging and differential interference contrast microscopy (Thorn, 2016). Manual reconstruction of 3D astrocyte models from aligned stacks of EM images is time-consuming and expensive, whereas automatic methods do not yet reach the level of precision required to capture all the astrocytic details. To date, EM is still the sole method capable of resolving cellular details in nanometer resolution (Calì et al., 2019). These features include astrocytic nanoscopic processes, synaptic contacts and vesicles, as well as intracellular organelles such as endoplasmic reticulum and mitochondria. Several methods for EM have been developed (Jacobs et al., 2010; Peddie & Collinson, 2014). Transmission EM (TEM) enables distinguishing features to a spatial resolution of around 1 nm. In order to reconstruct 3D volumes, planar images cut and imaged in series from tissue slices are registered and aligned. Serial section EM (SSEM) is a classic manual approach that utilizes TEM imaging (Harris et al., 2006). Many automated serial EM techniques have emerged that perform the reconstruction without human supervision (Peddie & Collinson, 2014). Focused ion beam scanning EM (FIB-SEM) is an automatic imaging technique that is considerably more effective than SSEM (Bushby et al., 2011). Serial block-face EM (SBF-SEM) is another automatic imaging technique that forms images utilizing backscatter detectors (Denk & Horstmann, 2004). FIB-SEM is intended for analyzing regional features whilst SBF-SEM is better suited for detailed analysis of larger features. The resolution of SBF-SEM is not enough for distinguishing nanometer-scale structures (Calì et al., 2016). It should be noted that treatments used for preparing tissue samples for EM imaging may create artifacts and influence the cell volume and surface area (Savtchenko et al., 2018).

#### Tracing

Skeletal models are obtained by tracing the branching morphology of a cell either manually or with automatic or semi-automatic methods from LM images and saving the 3D geometry and cross-sectional diameters of the neuronal arborization in a skeletal morphology format (Halavi et al., 2012) (Table 1). Most widely used commercial neuronal tracing software include Neurolucida (Glaser & Glaser, 1990) and FilamentTracer (https://imaris.oxinst.com/products/imaris-for-neuroscientists). Open-source tracing tools include Vaa3D (Peng et al., 2010, 2014), Neuromantic (Myatt et al., 2012), FARSIGHT (Luisi et al., 2011), Simple Neurite Tracer (Longair et al., 2011), and TREES toolbox (Cuntz et al., 2010). Tracing from EM images can be achieved with tools such as Ilastik (Kreshuk et al., 2011), TrakEM2 (Cardona et al., 2012), and webKnossos (Boergens et al., 2017).

#### Morphological Formats

Cell morphologies are represented in different formats, including skeletal models, polygonal surface mesh models, volumetric mesh models, and surface point cloud models (Table 3). To date, there is no standard file format for representing detailed whole-cell astroglial reconstructions, whereas for modeling neuronal reconstructions, an extensible markup language, MorphML under NeuroML, has been generated (Cannon et al., 2014; Crook et al., 2007; Gleeson et al., 2010). Astrocytic endfeet are particularly difficult to represent in any format commonly used for other central nervous system structures, since they cannot be represented by cyclic or acyclic graphs like neuronal or vascular morphologies, respectively (Abdellah et al., 2021). Skeletal models are useful for validating cell models and their connectivity patterns. Polygonal surface mesh models can be used for visualizing, for example, calcium concentrations and membrane potentials and performing particle-based stochastic reaction–diffusion simulations. Volumetric mesh models can be used for simulating light interaction with brain tissue and performing voxel-based stochastic reaction–diffusion simulations. Surface point cloud models are 3D shapes represented by sets of data points in space that can be used in some simulation tools to provide morphologies of detailed nanoprocesses.

**Table 3:** Examples of cell morphology formats. The descriptions and formats of different morphology types are presented

**Type**	**Type description**	**Format**	**Format description**
**Skeletal morphology**	Geometry of a cell represented as a hierarchical tree of linear segments with coordinates and radii for each cylinder	HOC	High Order Calculator
		JSON	JavaScript Object Notation
		MorphML/NeuroML	Extensible Markup Language Format
		Neurolucida ASC	ASCII Text Format
		Neurolucida DAT	Binary Format
		Neurolucida XML	Extensible Markup Language Format
		SWC	Stockley-Wheal-Cannon Format
**Polygonal surface mesh**	Cell surface represented with polygons, formed by vertices bounded by edges	ABAQUS	Abaqus Mesh Format
		BLEND	Blender File Format
		MSH	Gmsh ASCII Mesh Format
		NODE, FACE, ELE	TetGen’s Formats
		OBJ	Wavefront Object Format
		OFF	Object File Format
		PLY	Polygon File Format
		RAW	Raw Mesh Format
		STL	Stereolithography Format
		V3DS	Vaa3D’s Surface Format
**Surface point cloud**	Cell surface represented with coordinates	APO	Comma-separated Value Format
		DAT	Comma-separated Value Format
**Volumetric mesh**	Interior volume of a cell represented with polygons		Binary Formats
			Byte Formats

Skeletal morphologies are represented by hierarchical trees usually consisting of linear segments denoted with coordinates, cross-sectional diameters, connectivity links, and indexes for neurite types (soma, dendrite, axon, etc.) (Halavi et al., 2012). Skeletal models can be encoded in different formats from which open-source Stockley-Wheal-Cannon (SWC) format (Cannon et al., 1998) and HOC and closed-source Neurolucida DAT formats are the most common (Table 3). A limitation of both the SWC and HOC formats is that they represent neuronal somata as cylinders (Parekh & Ascoli, 2013). In contrast to the SWC and HOC formats, Neurolucida DAT format represents the soma with surface contouring (Glaser & Glaser, 1990). Reconstructing astrocytic stem trees with LM is similar to reconstructing neurons and can be implemented with the same tools and methods. In this case, tracing the astrocyte morphology includes only the stem tree, the larger branching structure of the astrocyte, whilst the endfeet and the cloudy structure formed by nanoprocesses are excluded. Astrocytic stem trees could also, provided that data is available, be constructed computationally based on quantitative data on stem tree branching patterns and branch diameters.

Polygonal surface meshes are obtained from EM images and consist of vertices, edges that connect the vertices, and polygonal faces that are bounded by neighboring edges (Botsch et al., 2007). The polygonal faces are usually composed of triangles or quadrilaterals. Standard file formats for storing polygonal surface meshes include polygon format (.ply), Wavefront object format (.obj), and stereolithography format (.stl). Multiple polygonal surface meshes can be stored in Blender as a blender file (.blend). It is important that the polygonized mesh preserves the surface area and volume of the original astrocyte, since the relationship between volume and surface area is highly important for astrocytic ionic signaling (Wu et al., 2019).

In addition to skeletal and polygonal surface mesh models, simulation tools utilize other formats including volumetric mesh models (Hepburn et al., 2012) and surface point cloud models (Savtchenko et al., 2018). Besides reaction–diffusion simulations, volumetric mesh models enable simulating light interactions within neural tissue. Volumetric mesh models represent the interior volume of a cell with polygons and can be stored either in binary or byte formats. Surface point clouds represent the cell surface with cartesian coordinates and can be stored from EM images in DAT or APO formats, for example. The DAT file format contains the surface coordinates of a morphology as separated by commas. The APO file format is a simple CSV format utilized by Vaa3D software.

#### Astrocyte Data in Literature and Online

Astrocytic skeletal morphologies are available at NeuroMorpho.Org database (Ascoli et al., 2007) in SWC format. Astrocytic bioimage data is also available in the Cell Centered Database (Martone et al., 2002). Other databases such as Allen Brain Atlas (Lein et al., 2007) and the BraVa database (Wright et al., 2013) contain data related to neurons and vasculature, respectively. Very few detailed whole-cell reconstructions of astrocytes exist and most of them have been implemented for cortical astrocytes (Calì et al, 2019; Coggan et al., 2018) and some for hippocampal astrocytes (Aten et al., 2022). For cerebellar astroglial cells, EM reconstructions of only Bergmann glial branches are available (Grosche et al., 1999).

#### Synthetic Morphologies

Computationally synthesizing cell morphologies provides an alternative to the expensive and time-consuming imaging and reconstruction of cell morphologies (Tables 1 and 2). Quantitative data available in the literature that could be utilized to build synthetic cell morphologies include measurements of cell dimensions, branching pattern, and connectivity (Calì et al., 2019; Lippman et al., 2008; Zisis et al., 2021). Skeletal models that consist of the main branching structure of the cell and their branch diameters can be constructed with the NEURON CellBuilder tool (Carnevale & Hines, 2006). TREES toolbox provides functionality for generating synthetic axonal and dendritic trees. In addition, algorithmically synthesized astrocyte reconstructions are available in the NGV Portal (Abdellah et al., 2021; Calì et al., 2019; Zisis et al., 2021). The approach adapted by Abdellah et al. (2021) in NeuroMorphoVis for constructing high-fidelity astrocytic volumetric meshes was to use metaballs in Blender. The implicit surface formed by metaballs was converted into a polygonal surface mesh from which a volumetric mesh was produced. The advantage of metaballs is that it allows merging segments of the cell morphology seamlessly into one morphology with no self-intersecting segments. In contrast to neurons which are acyclic, vasculature and possibly some astrocytes are cyclic and thus benefit from the metaball approach.

### Validation

After construction, the validity and accuracy of the resulting morphologies can be evaluated with different tools (Tables 1 and 2). The constructed morphologies should capture the characteristics of the biological structures they aim to reproduce. Validation of astrocytic morphologies includes visualization, optimization for different purposes, and assessing morphometrics.

#### Visualization

Visualizations range from 2D images to 3D and virtual reality simulations. Existing computational tools allow visualizations of different types of morphological formats in different abstraction levels. Abstractocyte enables visualizing astrocytes together with neurons with independently chosen abstraction levels (Mohammed et al., 2018) (Table 1). Exploring the intracellular distribution of structures such as mitochondria or glycogen granules can be achieved visually with Abstractocyte (Mohammed et al., 2018). Other non-Blender tools for visualization include, for example, Open Source Brain (Gleeson et al., 2019) (Table 1). Blender-based tools for visualizing include, for example, CellBlender (Kerr et al., 2008), NeuroMorph (Jorstad et al., 2015, 2018), NeuroMorphoVis, Py3DN (Aguiar et al., 2013), and blenderNEURON (https://blenderneuron.org/) (Table 2). In addition, Blender has been utilized for creating 3D visualizations of neural cells, including astrocytes, which have been explored within an immersive virtual reality environment CAVE (Calì et al., 2016, 2019). A complete neuro-glia-vascular ensemble has been reconstructed with Blender (Coggan et al., 2018), as well as a detailed ultrastructural astrocytic arborization including intracellular structures and connections with other astrocytes and synapses (Aten et al., 2022). Of the tools presented in Table 2, cellPACK (Johnson et al., 2015) and ePMV (Johnson et al., 2011) can be run on any of the platforms; Cinema 4D, Maya, and Blender.

#### Optimization

Depending on the intended usage of the morphologies, their different properties may need to be adjusted. The specific requirements may include detecting and repairing artifacts, mesh decimation, boundary marking, and curvature estimation. Blender-based tools for assessing and repairing neuronal polygonal surface and volumetric meshes include, for example, BlendGAMer (Lee et al., 2020) (Tables 2). Our CellRemorph toolkit allows automatic removal of disconnected segments from the main morphology and decimating the polygonal surface mesh for more efficient simulations.

#### Morphometrics

Computational tools for morphometric measurements allow assessing crucial astrocytic properties such as the volume, surface area, length, connectivity, spatial distribution of intracellular molecules, and distances between different structures (Tables 1 and 2). These morphological parameters influence the diffusion of different intracellular molecules and ions. Several of the Blender add-ons, including our CellRemorph toolkit, perform morphometric measurements.

### Transformation

The route from images to 3D models that are usable in different types of simulations requires specialized computational tools for transforming them into different formats (Tables 1 and 2). Each of the four categories from simpler to more complex, including skeletal, surface point cloud, polygonal surface mesh, and volumetric mesh models, can be represented in multiple formats. Transformations can be executed between different formats in one category and between different categories.

#### Skeletal Models

Transforming skeletal neural models between different skeletal formats is affected mainly by nomenclature and is relatively easy in most cases, and automatic converters exist for this purpose (Table 1). The most extensive converter is NLMorphologyConverter available at NeuronLand (http://neuronland.org/NL.html) and focused converters also exist. As an example, an SWC-formatted morphology can be transformed into HOC format and vice versa quite easily with the NLMorphologyConverter. In addition, NEURON automatically converts imported SWC to HOC and hoc2swc can be used to perform the reverse conversion (https://github.com/JustasB/hoc2swc). NeuronLand also contains a listing of all the formats for either 3D, 2D, or 1D representations of skeletal models. SWC-formatted morphologies can be transformed into NeuroML using, for example, Open Source Brain, neuroConstruct (Gleeson et al., 2007), and Cvapp for NeuroMorpho.org (https://github.com/pgleeson/Cvapp-NeuroMorpho.org/), an updated version of the original Cvapp (Cannon et al., 1998). neuroConstruct is also able to transform other skeletal formats into NeuroML. pyNeuroML includes utility for transforming morphologies in NeuroML format into SWC (https://github.com/NeuroML/pyNeuroML). Skeletal models can also be reconstructed from polygonal surface meshes in HOC format using VolRoverN (Edwards et al., 2014).

#### Polygonal Surface Meshes

Transforming skeletal models into polygonal surface meshes can be more challenging, and various approaches have been developed (Tables 1 and 2). Applications ranging from simple visualizations to complex simulations pose different requirements on how these meshes have to be constructed. Polygonal surface mesh models have to be often decimated by adjusting their tessellation levels in order to reduce their computational burden for simulations and optimize them for different purposes (Lee et al., 2020). In general, meshes used for visualization purposes do not need to be non-intersecting (i.e., watertight) and can be highly tessellated, while meshes used for simulations should be watertight with reduced tessellation for lighter-weight simulations. Blender by default includes a subdivision library that enables reducing the tessellation levels of meshes without significantly affecting their surface areas or volumes. The cell bodies that are often represented as cylinders in the skeletal models need to be reconstructed in a more realistic manner as polygonal surface meshes. Tools for transforming skeletal morphologies into polygonal surface meshes with realistic somata include, for example, SWC Mesher developed by the MCell team (https://github.com/mcellteam/swc_mesher), Neuronize (Brito et al., 2013), NeuroTessMesh (Garcia-Cantero et al., 2017), and NeuroMorphoVis (Tables 1 and 2). For simple visualization and manipulation in Blender, SWC2Blender (https://github.com/MartinPyka/SWC2Blender) transforms SWC-formatted morphologies into Blender Bezier curve representations. Our CellRemorph add-on allows the direct transformation of nanoprocesses selected from surface point clouds into polygonal surface meshes.

#### Volumetric Meshes

Polygonal surface meshes can be transformed into volumetric mesh models via tetrahedralization. Tetrahedralization can be implemented with generic tools, such as TetGen (Si, 2015), QUARTET (Labelle & Shewchuk, 2007), CGAL (Boissonnat et al., 2002), and TetWild (Hu et al., 2018), as well as with neuroscience-specific tools that provide functionality for tetrahedralization, such as VolRoverN and NeuroMorphoVis. Except from TetWild, all these tools require the polygonal surface mesh to be smooth, continuous, and watertight. While many of these tools are commonly used for transforming neural morphologies into volumetric mesh models, Tables 1 and 2 include only those tools that are specifically aimed towards tetrahedralization of neural morphologies. NeuroMorphoVis includes a custom tool for creating volumetric mesh models by first creating a volumetric shell of the polygonal mesh via surface voxelization that uses conservative rasterization, and subsequently filling the intracellular space of the neuron utilizing a solid voxelization method such as flood-filling algorithm (Abdellah et al., 2018).

#### Surface Point Clouds

None of the above published tools provides utility for transforming whole polygonal surface meshes into adjustable surface point clouds. This functionality is important because some simulation tools need morphologies in surface point cloud formats. To address this challenge, we present the CellRemorph toolkit that can be used in combination with other utilities and tools available by default in Blender or provided by other Blender add-ons (Table 2).

### Subdivision

Subdividing astrocyte morphologies allows assessing morphometric measurements from cell segments and performing different types of simulations. It can also be useful for visualization as well since astrocyte morphologies are so complex that observing whole-cell morphologies in detail may occlude visualization of some structures. Visualizing, validating, and transforming cell segments is also computationally less burdensome than with the whole-cell morphology. In addition, cell segments can be easier to transform into certain formats than whole-cell morphologies. CellRemorph includes two subdivision tools, one for selecting nanoprocesses and the other one for slicing the morphology into segments equal in volume or surface area (Table 2). Another Blender add-on, CellBlender module NeuropilTools, allows subdividing dendritic spines into separate components, such as the neck and head (Bartol et al., 2015).

### Simulation

Computational modeling of astrocytes and simulations of their functions can be performed utilizing morphologies which are either in skeletal, surface point cloud, polygonal surface mesh, or volumetric mesh formats. While the cylinder representation of skeletal morphology formats can be suited for capturing the important features of neuronal morphologies, astroglial morphologies are more complex and characterized by high surface-to-volume ratios and not as well represented by cylinders. A commonly used simulation platform that utilizes skeletal models is NEURON (Carnevale & Hines, 2006), which has been extended in the ASTRO tool (Savtchenko et al., 2018) to accommodate detailed whole-cell modeling of astrocytes. The approach adapted in ASTRO is to represent the stem tree of an astrocyte as a skeletal model and populate it algorithmically with a simplified yet function-preserving representation of the astrocytic nanoscopic geometry. Creating a realistic nanoscopic geometry for the astrocyte with ASTRO requires a detailed 3D EM of multiple astrocyte sections in surface point cloud format. ASTRO Nanogeometry module (Savtchenko et al., 2018) allows selecting nanoprocesses from a point cloud formatted morphology and converts them into cylindrical shapes to be used in ASTRO simulations. To our knowledge no other astrocyte-specific simulation tools that take the detailed astrocyte morphology into account yet exist, but MCell (Kerr et al., 2008) and STEPS (Denizot et al., 2019; Hepburn et al., 2012) are examples of simulation platforms that are useful for simulating morphologically detailed astrocytes, as well. Both MCell and STEPS enable stochastic reaction–diffusion simulations, but the former requires morphologies in polygonal surface mesh format and the latter in volumetric mesh format.

## The CellRemorph Toolkit

The CellRemorph toolkit is written in Python 3.5 and is freely available in GitHub (https://github.com/lauraketo/CellRemorph) together with a manual and instructional video to be used as Blender add-on. The CellRemorph toolkit can be used on morphologies that are either in the format of surface coordinates (surface point clouds) or polygonal surface meshes reconstructed from a series of images. The CellRemorph toolkit includes three tools: one for selecting nanoprocesses from polygonal surface meshes or surface point clouds, second for transforming polygonal surface meshes into surface point clouds and vice versa, and third for slicing a morphology into segments equal either in volume or surface area (Table 2). Figure 1b depicts the CellRemorph toolkit opened in Blender with the option “3 Slicer” selected. We have tested and evaluated the functionality of our tools with different astrocyte morphologies found in databases. These morphologies have complex arborizations with high surface-to-volume ratios characteristic to astrocytes. Details of all the tools are described below and in the manual available in the Supplementary Material and GitHub.

### Selector

**Fig. 2:**
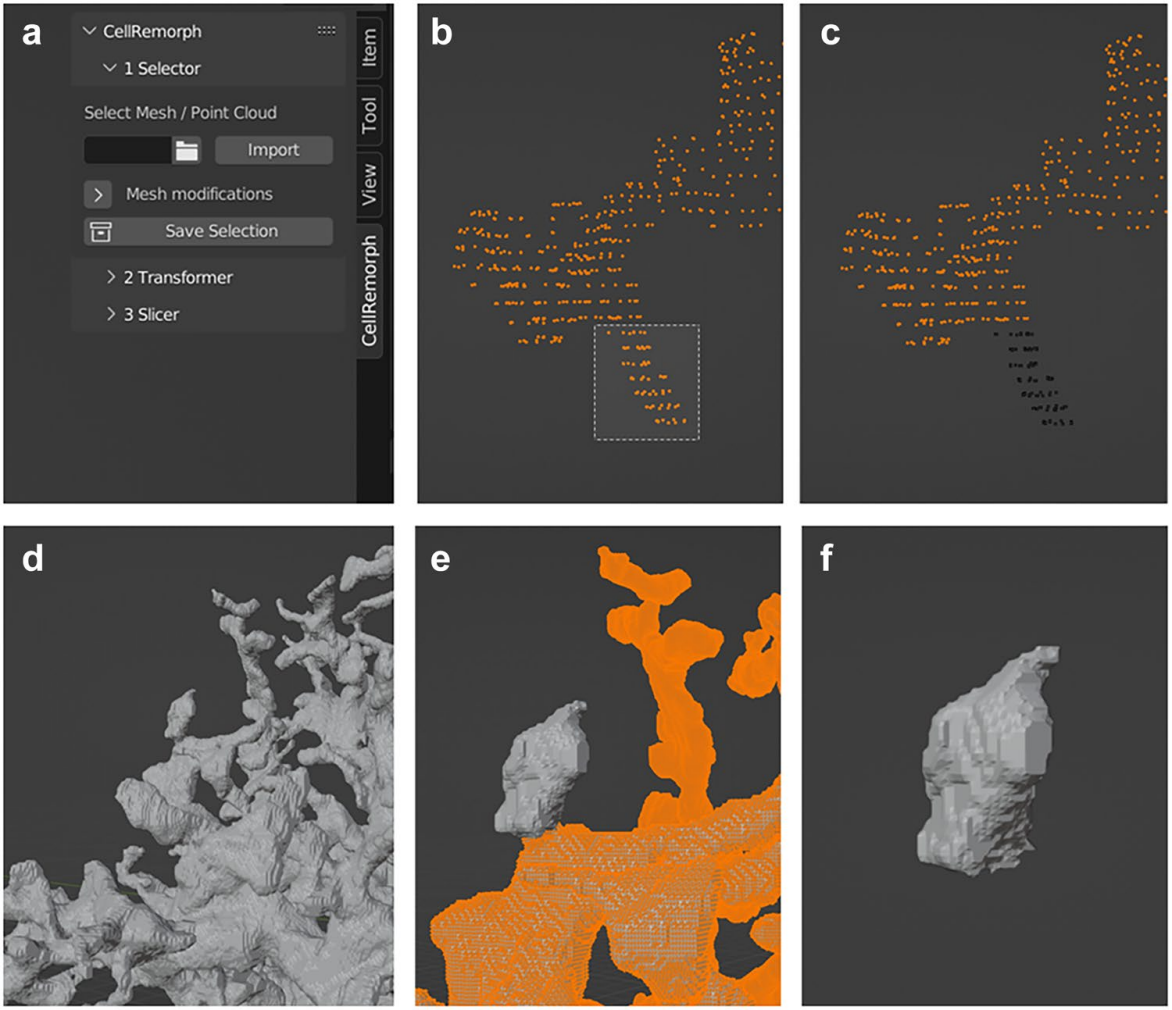
Selecting nanoprocesses from surface point clouds and polygonal surface meshes with the first tool. **a** The user interface of CellRemorph with the “Selector” subpanel opened. **b** A nanoprocess selected from the astrocyte morphology which is in surface point cloud format obtained from ASTRO (https://github.com/LeonidSavtchenko/Astro/blob/master/nanoGeometry/testshape.dat; Savtchenko et al., 2018). **c** The selected nanoprocess in b displayed in black is separated from the rest of the point cloud. **d** Part of the mesh formatted astrocyte morphology presented in Fig. 1a, obtained from the NGV Portal (https://bbp.epfl.ch/ngv-portal/anatomy/reconstruction-data/; Abdellah et al., 2021; Calì et al., 2019; Zisis et al., 2021). **e** The nanoprocess selected from the astrocyte morphology in d. **f** The same nanoprocess as in e, displayed separately from the rest of the mesh

The first tool allows for selecting nanoprocesses from surface point clouds or polygonal surface meshes and saving them into separate surface point cloud or polygonal surface mesh structures, respectively (Fig. 2a). Other tools, such as NeuroMorph, allow selecting segments from polygonal surface meshes but not from point clouds. The CellRemorph toolkit automatically calculates the morphometrics of shapes selected from polygonal surface meshes, including volume and surface area, and saves them. In addition, the mesh modification subpanel allows changing the tessellation level of the original polygonal surface mesh and removing any disconnected segments. Figure 2b-c showcases the selection of a nanoprocess from a surface point cloud formatted hippocampal area CA1 astrocyte obtained from ASTRO (https://github.com/LeonidSavtchenko/Astro/blob/master/nanoGeometry/testshape.dat; Savtchenko et al., 2018). Figure 2d-f showcases the selection of a nanoprocess from a synthesized astrocyte polygonal surface mesh obtained from the NGV Portal (https://bbp.epfl.ch/ngv-portal/anatomy/reconstruction-data/; Abdellah et al., 2021; Calì et al., 2019; Zisis et al., 2021) and saved as a mesh.

### Transformer

**Fig. 3:**
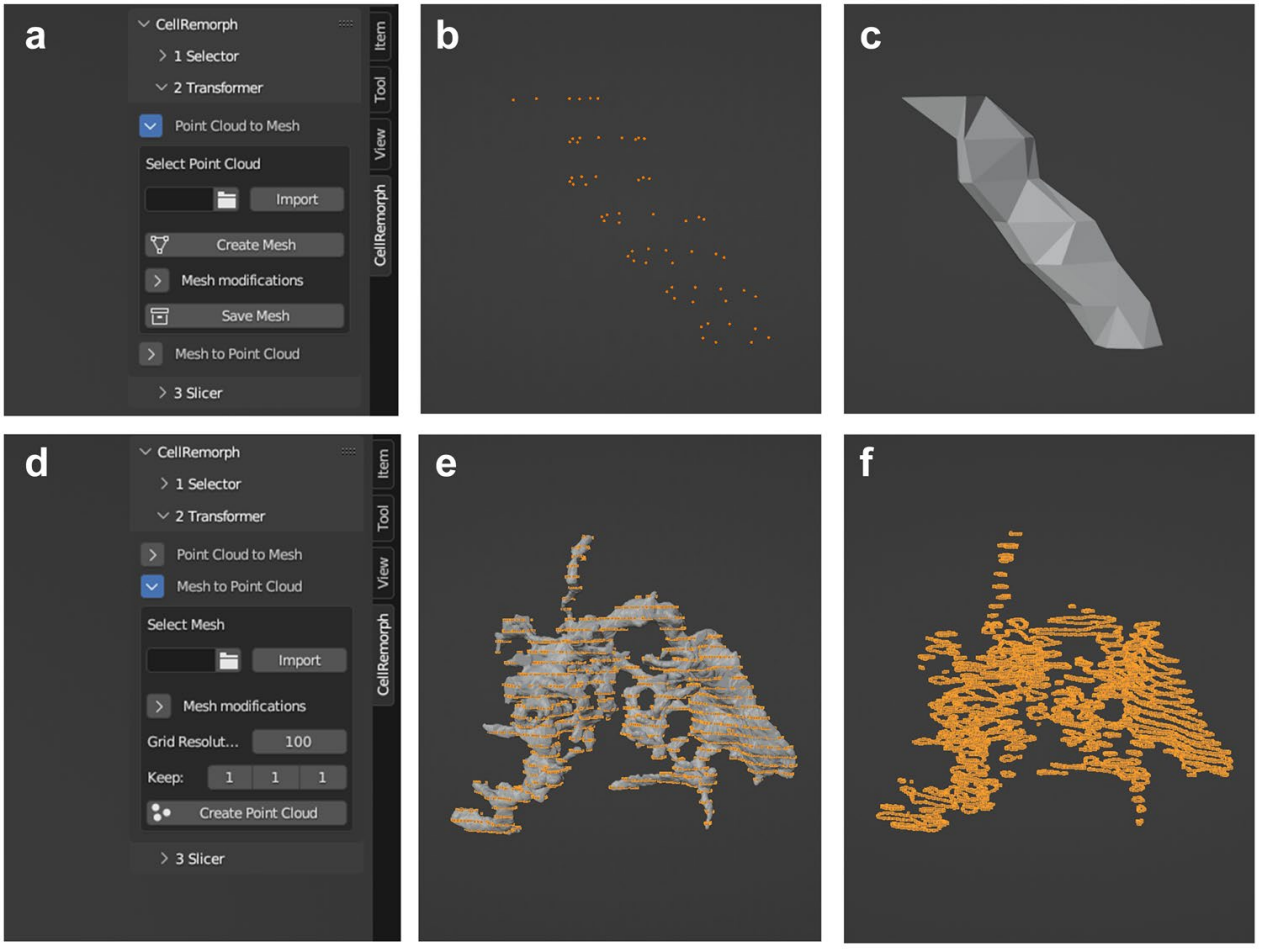
Converting surface point clouds to polygonal surface meshes and vice versa with the second tool. **a** The user interface of CellRemorph with the “Transformer” subpanel and the option “Point Cloud to Mesh” opened. **b** The nanoprocess selected from the point cloud with the first tool in Fig. 2c. **c** The nanoprocess in b transformed into a mesh. **d** The “Transformer” subpanel and the option “Mesh to Point Cloud” opened. The parameter “Grid resolution” defines the number of surface points along the longest axis of the mesh. The parameter “Keep” denotes for each axis every nth circumvent of points to keep. With the default setting (x = 1, y = 1, z = 1) every circumvent of points is kept in each direction. **e** A point cloud created from a part of the astrocyte morphology presented in Fig. 1a, obtained from the NGV Portal (https://bbp.epfl.ch/ngv-portal/anatomy/reconstruction-data/; Abdellah et al., 2021; Calì et al., 2019; Zisis et al., 2021) with the following parameters: grid resolution 200 and keep x/y/z as 1/1/6. **f** The same astrocyte morphology as in e but only showcasing the created point cloud

The second tool enables transforming morphologies from surface point clouds into polygonal surface meshes and vice versa (Fig. 3a, d). These transformations allow utilizing whole-cell morphologies or cell segments in simulation tools that require them in either of these formats as well as performing morphometric calculations. To our knowledge, no previous tools for these purposes have been developed. Transforming polygonal surface meshes into surface point clouds can be executed on whole-cell morphologies as well as any cell segments (Fig. 3e). The point clouds created with the second tool consist of points aligned in regular intervals directly on top of the original mesh surfaces, with precision adjustable in the user interface along any of the axes. Figure 3e and f showcases a part of the astrocyte polygonal surface mesh obtained from the NGV Portal transformed into a point cloud. Transforming point clouds into polygonal surface meshes can be performed only on cell segments, such as nanoprocesses, that are represented by loops of points aligned in two coordinate axes (Fig. 3b). Nanoprocesses and other cell segments that fulfill this requirement can be selected from point clouds by utilizing the first tool in the CellRemorph toolkit (Selector). The surface meshes created from the point clouds with the second tool consist of triangular polygons. The morphometrics of point clouds transformed into surface meshes, including volume and surface area, are automatically calculated and saved. Figure 3b showcases the nanoprocess selected from the point cloud with the first tool in Fig. 2c, and Fig. 3c showcases the nanoprocess in Fig. 3b transformed into a polygonal surface mesh.

### Slicer

**Fig. 4:**
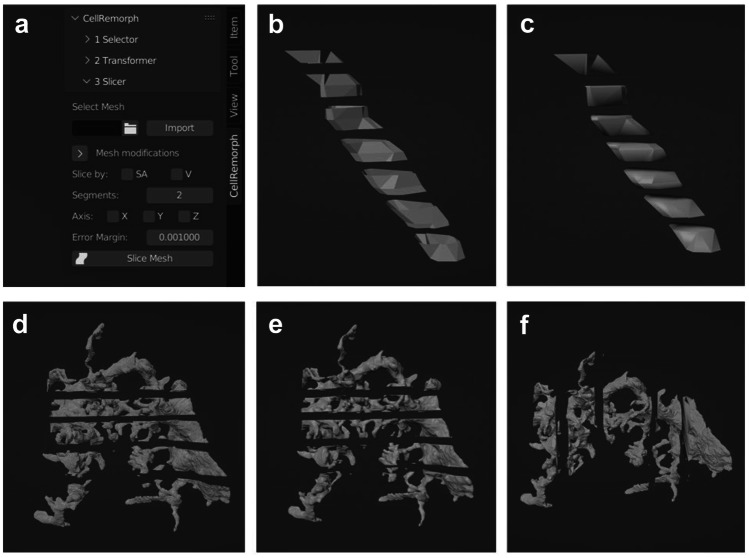
Slicing astrocyte morphologies into segments with equal surface areas or volumes with the third tool. **a** The user interface of CellRemorph with the “Slicer” subpanel opened. The parameter “Slice by” allows choosing to slice either by surface area (SA) or volume (V). The parameter “Segments” determines how many segments the morphology is sliced into. The parameter “Axis” defines the axis by which the shape is sliced. The parameter “Error Margin” defines how precisely the surface areas / volumes of the resulting segments correspond to each other. **b** The nanoprocess, selected with the first tool in Fig. 2c and transformed into a mesh with the second tool in Fig. 3c, sliced into seven segments of equal surface area along the z-axis with an error margin of 0.00001. **c** The same nanoprocess that was used in b sliced into seven segments of equal volume along the z-axis with an error margin of 0.00001. **d** The same astrocyte morphology as in Fig. 3e obtained from the NGV Portal (https://bbp.epfl.ch/ngv-portal/anatomy/reconstruction-data/; Abdellah et al., 2021; Calì et al., 2019; Zisis et al., 2021) sliced into five segments of equal surface area along the z-axis with an error margin of 0.001. **e** The same astrocyte morphology that was used in d sliced into five segments of equal volume along the z-axis with an error margin of 0.001. **f** The same astrocyte morphology that was used in d and e sliced into five segments of equal volume along the x-axis with an error margin of 0.001

The third tool is for slicing surface mesh structures along any of the three axes to produce a desired number of segments equal either in volume or surface area (Fig. 4a). In addition, the mesh modification subpanel allows changing the tessellation level of the original mesh and removing any disconnected segments. While other tools, such as NeuroMorph, allow morphometric calculations, the slicing into segments equal in volume or surface area is provided only in CellRemorph. The main difference between slicing by volume and surface area is that slicing by volume encloses the ends of the sliced structures with surfaces while slicing by surface area leaves them hollow. Figure 4b displays the slicing of the cell structure shown in Fig. 3c into seven segments equal in surface area, whereas in Fig. 4c they are equal in volume. Figure 4d showcases the slicing of the morphology in Fig. 3e into five segments equal in surface area, whereas Fig. 4e showcases five segments equal in volume. Figure 4f showcases the slicing into five segments equal in volume in different direction.

### Exporting Results

CellRemorph saves the point cloud coordinates into a DAT file containing the XYZ surface coordinates. The surface mesh structures are saved as 3D mesh format OBJ. Both the DAT and OBJ files are stored in new folders. In addition, CellRemorph produces a report in DAT format which contains the elapsed times and applied parameters. Depending on the tool, other values, such as the calculated surface areas and volumes, are provided.

## Discussion

Only through combining data from experimental studies with morphologically detailed whole-cell astrocyte models can we study and understand in detail the roles of astrocytes in health and disease. Simulating calcium waves realistically requires models that capture the complex nanoscopic architecture of astrocytes and allows probing astroglial functions difficult to study experimentally. Most of the existing astrocyte models, however, are single-compartmental models that can only provide a simplification of the real system (Manninen et al., 2018). Modeling astrocytes in morphologically detailed manner is intrinsically challenging because imaging of astrocytes is both time-consuming and expensive, only a few whole-cell astrocyte morphologies exist in databases, not all the formats of astrocyte morphologies are suitable for morphologically detailed astrocyte simulations, different simulation tools require distinct formats for cell morphologies, and computational tools specific to astrocytes are rare.

Astrocytes are known for their numerous nanoscopic processes which are highly relevant for their functioning (Schiweck et al., 2018; Verkhratsky & Nedergaard, 2018). Light microscopy, which can only distinguish the larger branches of astrocytes and is utilized for creating skeletal models, is not enough to capture all the complex features of astrocyte morphologies. To date, the only imaging method able to capture all the astrocytic morphological details is electron microscopy, which is both more time-consuming and expensive than light microscopy. For this reason, only a limited number of detailed whole-cell astrocyte morphologies exist that could be utilized in simulations. Commonly used formats for representing cell morphologies provided by electron microscopy are surface point cloud and polygonal surface mesh models from which volumetric mesh models can be created. Each of the above four model categories can be represented by many file formats (Table 3) since no standard file formats exist for representing astrocyte reconstructions in the different categories. To reconstruct cell morphologies, enable transformation of morphologies into different formats, either under the same category or between different categories, and validate and visualize them, a variety of computational tools have been developed, many of which are based on Blender (Tables 1 and 2).

To date most of the Blender add-ons relevant for neuroscience are aimed for manipulating cellular-scale neuronal morphologies, but there are molecular-level tools BioBlender (Andrei et al., 2012) and ePMV (Johnson et al., 2011), as well as a cell network level tool PAM (Pyka et al., 2014) and brain vasculature tool VessMorphoVis (Abdellah et al., 2020) (Table 2). While only the CellRemorph toolkit presented in this study and NeuroMorphoVis (Abdellah et al., 2018, 2021) include utility specifically aimed for astrocytes as well as neurons, tools aimed for neuronal cells can be useful for manipulating astrocyte morphologies as well. CellRemorph provides three tools for manipulating astrocyte morphologies (Figs. 1, 2, 3, and 4). The transformer tool in the CellRemorph toolkit facilitates the creation of high-fidelity polygonal surface meshes from surface point clouds and vice versa (Fig. 3). This tool is needed because some simulation tools require morphologies in point cloud format and some others in surface mesh format, and none of the previous tools provide this functionality. Currently the transformation from surface point clouds to surface mesh format is possible only for nanoprocesses consisting of points arranged into loops along their length. This functionality could be extended to allow the transformation of different, more complex shapes in the future. The accuracy of the transformation from surface meshes to point clouds could potentially be improved by adjusting the locations of the surface points to be better aligned with the surface of the mesh. Since astrocytic surfaces are characterized by complex nanoscopic processes, the selector tool provided in the CellRemorph toolkit enables selecting nanoscopic processes from astrocyte morphologies in surface point cloud and polygonal surface mesh formats (Fig. 2). With one of the previous tools, NeuroMorph (Jorstad et al., 2015, 2018), it is possible to select segments from surface meshes but not from point clouds. The slicer tool provided in the CellRemorph toolkit enables slicing the astrocyte morphology along any direction into desired number of segments equal in volume or surface area (Fig. 4). This tool is important because the volume and surface area are essential parameters influencing the propagation of molecules and ions across the astrocyte morphology and none of the previous tools can slice morphologies into segments equal in volume or surface area. The CellRemorph toolkit presented in this study could be improved in the future by slicing the morphology in such a way that the continuity of the resulting segments is preserved.

Since the need for realistic astrocyte modeling is increasing, the computational neuroscience community needs more whole-cell astrocyte morphologies to be available in the open-access repositories, more astrocyte-specific computational and simulation tools developed, for example, by extending previous tools made for neurons, and a standard file format for representing astrocyte morphologies, for example by extending MorphML and NeuroML (Cannon et al., 2014; Crook et al., 2007; Gleeson et al., 2010), to ease the utilization of different simulation tools in modeling. The tools in the CellRemorph toolkit, while mainly directed for preparing astrocyte morphologies for reaction–diffusion simulations, can be useful for manipulating any 3D morphologies and extend the pool of computational tools aimed for neuroscience studies available in Blender.

## Conclusions

The path from experimental data into morphologically detailed computational modeling and simulations requires novel methods for processing imaging data to accommodate different simulation tools. Astrocyte morphologies, characterized by an elaborate surface abundant with nanoscopic protrusions, are particularly challenging for simulation projects. Novel computational tools are needed to reconstruct and manipulate complex astrocyte morphologies, among other brain cells and structures. The functionality of the CellRemorph toolkit lies in the middle of the trajectory from experimental data to computational modeling and simulations, providing tools for transforming and subdividing astrocyte and other cell morphologies previously extracted from biological data into formats and segments suitable for different types of simulation tools and approaches.

## Information Sharing Statement

The software is available in GitHub (https://github.com/lauraketo/CellRemorph) under the GNU General Public License together with the manual and instructional video to learn how to use the software. The software manual is also available in the Supplementary Material.

## Supplementary Information

Below is the link to the electronic supplementary material.

Supplementary file1 (PDF 812 KB)
