# Supplementary material for: CellRemorph: A Toolkit for Transforming, Selecting, and Slicing 3D Cell Structures on the Road to Morphologically Detailed Astrocyte Simulations
Source: Neuroinformatics. 2023 May 3;21(3):483–500. doi: 10.1007/s12021-023-09627-5 (PMC10406679; doi:10.1007/s12021-023-09627-5)
Supplement: Supplementary file 1 — Supplementary file1 (PDF 812 KB) [file 12021_2023_9627_MOESM1_ESM.pdf]

The CellRemorph Toolkit

Contents

1. Starting 1
2. The Tools 3
   2.1 Selector 3
   2.2 Transformer 5
   2.3 Slicer 7
3. How the surface point clouds are transformed into meshes 8
4. References 9

Astrocyte morphologies are highly complex and defined by many small protrusions called nanoprocesses. This toolkit provides tools that facilitate the study and utilization of astrocyte morphologies for a variety of different applications. The CellRemorph toolkit includes tools for selecting nanoprocesses from a polygonal surface mesh or a surface point cloud, converting nanoprocesses selected from a surface point cloud into polygonal surface meshes, generating equidistant point clouds onto mesh surfaces, and slicing polygonal mesh structures either by surface area or volume. The tools are intended for astrocyte and other cell morphologies that are in a surface point cloud (.dat) or polygonal surface mesh (.obj) format. The DAT file format contains the XYZ surface coordinates of a morphology as separated by commas. The XYZ coordinates for each surface point are separated by whitespaces. The OBJ file format contains the surface geometry of a 3D model, including the position of vertices, faces, and edges.

The surface point cloud utilized in tool 1 was downloaded from https://github.com/LeonidSavtchenko/Astro/blob/master/nanoGeometry/testshape.dat (Savtchenko et al., 2018). The example morphology utilized in tools 2 and 3 is a nanoprocess selected from the surface point cloud with tool 1 and converted into a mesh with tool 2. Additional morphologies can be found in databases and publications (see, e.g., http://neuromorpho.org/, Ascoli et al., 2007). The cell morphologies in the NeuroMorpho.Org database are in the SWC format, which defines the three dimensional structure of a cell by specifying the type, coordinates, radius, and parent compartment for each cell compartment. SWC formatted cell morphologies can be converted into polygonal surface meshes with tools such as the SWC Mesher (https://github.com/mcellteam/swc_mesher) or NeuroMorphoVis (https://github.com/BlueBrain/NeuroMorphoVis, Abdellah et al., 2018).

1. Starting

Download the CellRemorph.zip file (no need to unzip it!). Download Blender 3.4 from https://www.blender.org/download/ and follow their instructions to install it.

Launch blender and go to edit/preferences (Figure 1A). In the preferences window, find Add-ons from the left panel and click Install on the top (Figure 1B). Find CellRemorph.zip-file from the folder where you

downloaded it and click “Install Add-On”. The add-on should be displayed on the list now. You can write the name “CellRemorph” to the search panel to easily find it. Click the checkbox located next to “Mesh: CellRemorph” to enable the add-on (Figure 1C). Close the preferences window. In the main view, press the key “N” and a panel opens on the right. CellRemorph can be found from the bottom of the narrow horizontal part of the sidebar on the right, click it to open it (Figure 1D). The usage of each tool in the add-on is presented in the following sections separately.

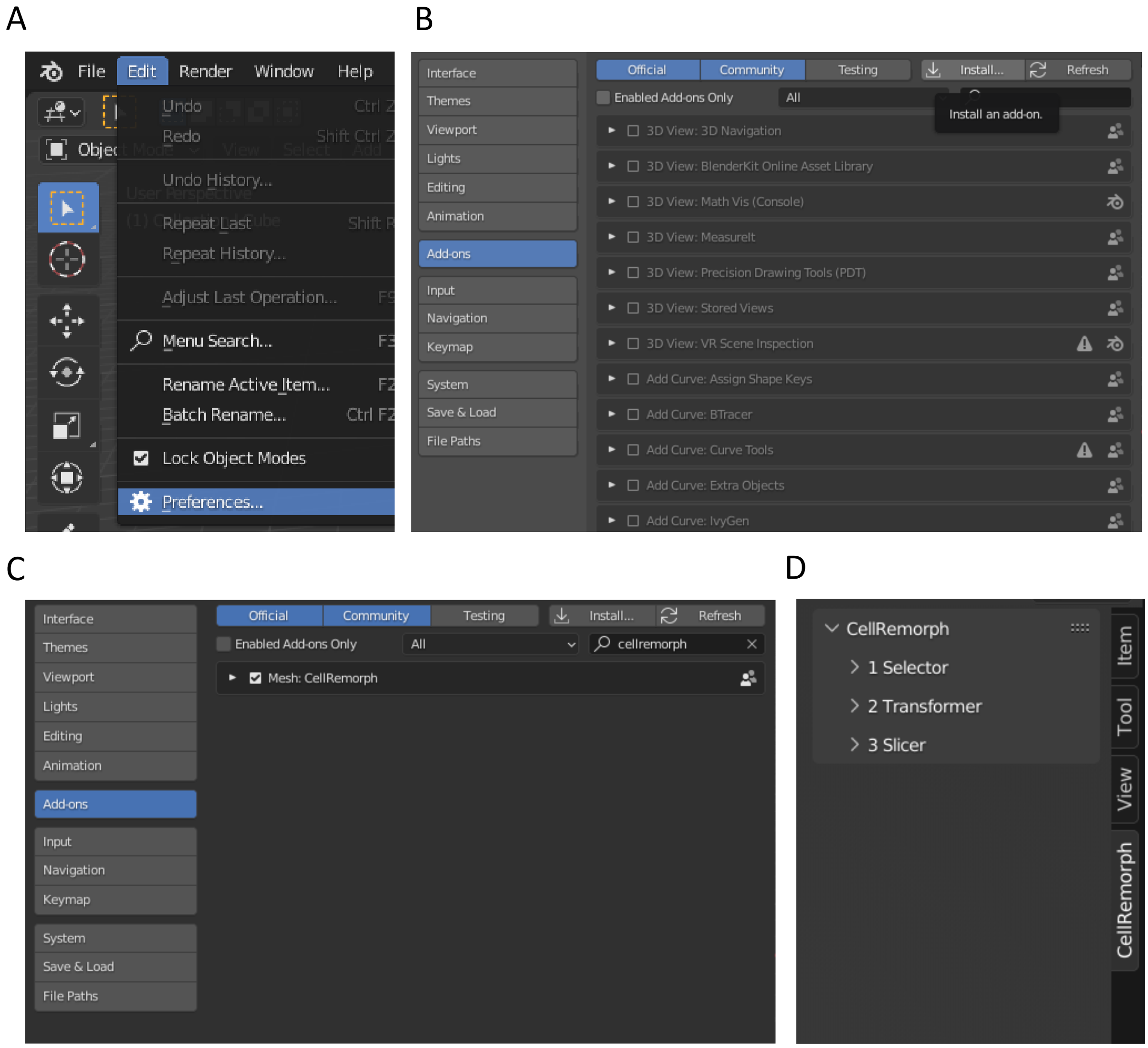

Figure 1. A) Go to edit/preferences to install the add-on. B) Install the add-on. C) Enable the add-on. D) The CellRemorph add-on can be found in the narrow horizontal part of the sidebar on the right; click it to open it.

2. The Tools

2.1 Selector

This tool allows for selecting nanoprocesses from surface point clouds or polygonal surface meshes and transforming them into separate surface point cloud or polygonal surface mesh structures, respectively.

Open the “Selector” subpanel and click the folder icon to select a polygonal surface mesh (.obj) or a surface point cloud (.dat) (Figure 2A). After selection click “Import” to import the mesh or point cloud into Blender. A selected mesh can be modified before selecting nanoprocesses. Clicking “Mesh modification” opens a subpanel with options for remeshing (you can specify the octree depth and scale of remeshing, visualize the result by clicking “Remesh”, and apply it by clicking “Apply”) and removing segments that are disconnected from the main shape (option “Clean Mesh”). The two parameters for remeshing, the octree depth and scale, define the resolution of the mesh; for both parameters higher values reproduce the mesh in finer detail. A point cloud imported into Blender is displayed in Figure 2B. The shape can be rotated by pressing the key “R”, moving the mouse, and clicking the left mouse button. To specify rotation along an axis, click the key “R” and then “X”, “Y”, or “Z”. The shape can be zoomed by scrolling the mouse wheel. In order to use the Blender box-tool to select nanoprocesses, click on “Toggle X-Ray” that ensures that hidden points are also selected (Figure 2C). Draw a box around the nanoprocess with the left mouse button (Figure 2D). By releasing the mouse button, the nanoprocess is selected. If the shape is not completely selected, additional points can be selected by pressing and holding “shift” while using the box-selection method described above. Nanoprocesses selected from a mesh or point cloud are saved as meshes (.obj) or point clouds (.dat), respectively. Click “Save Selection”, and the selection is immediately converted into a separate mesh or a point cloud displayed in gray or black, respectively. After saving one selection, more nanoprocesses can be selected and saved in a similar manner. For shapes selected from a mesh, the volume and surface area of each shape are also calculated and saved into an output-file (.dat). The Blender outliner context menu (in the upper right corner) contains the object and the selected shapes within a subcollection under the “Scene Collection”. To rotate or move both the original object and the selected shapes, change to the “Object Mode” first. The menu for changing the mode is located at the upper left corner in Blender (Figure 2E). Once in the “Object Mode”, press the key “A”, after which all of the shapes can be rotated or moved concurrently. In order to select more shapes, deselect other shapes by clicking the original object name in the outliner context menu and change back to the “Edit Mode”. Figure 2F showcases the selected nanoprocess. The saved meshes and/or point clouds can be found in subdirectories within your input directory.

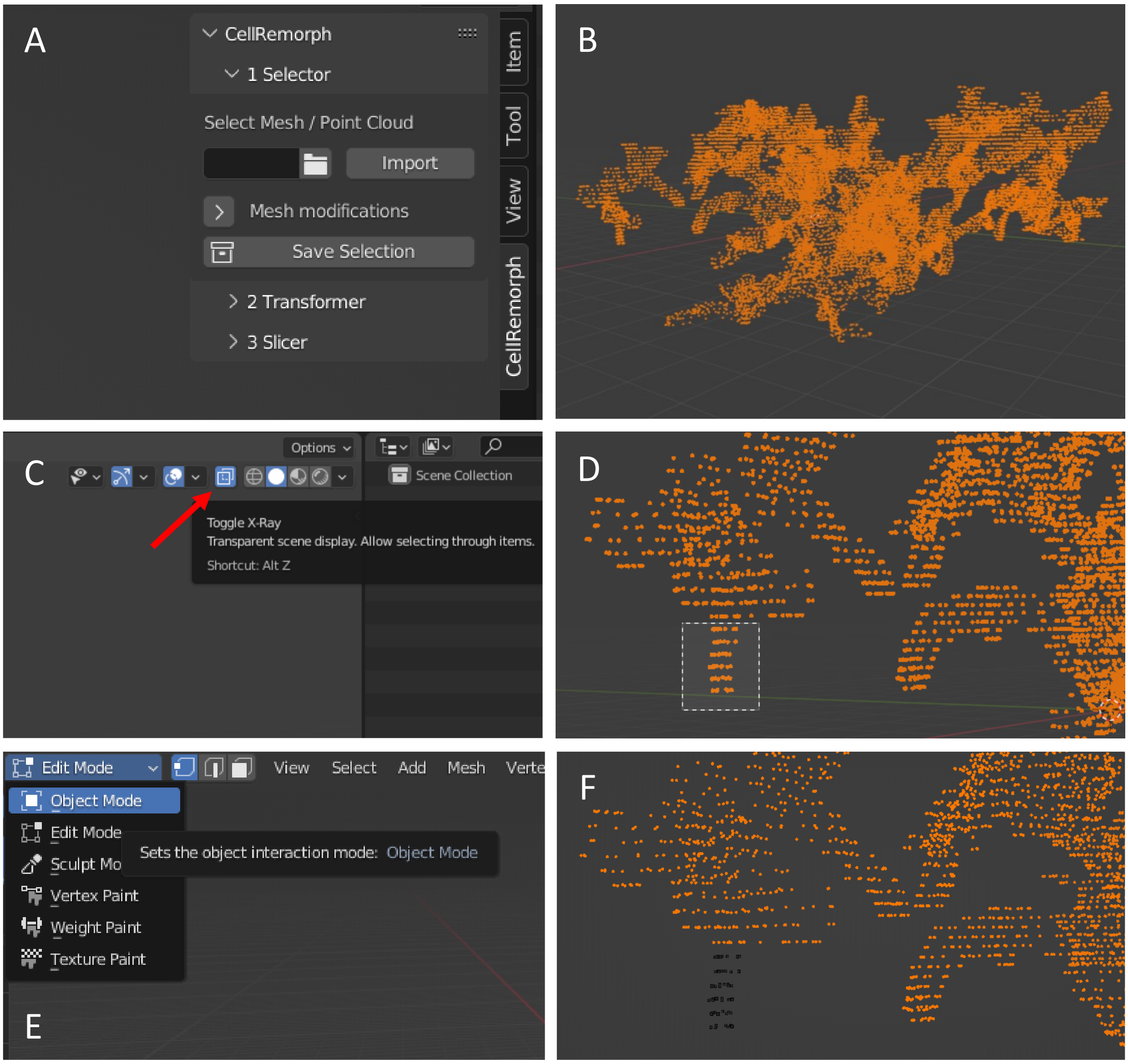

Figure 2. A) The "Selector" subpanel opened. B) The surface point cloud, taken from Savtchenko et al. 2018 (ASTRO tool), imported into Blender. C) Click on the "Toggle X-Ray". D) Selection of the nanoprocess with the Blender box-tool. E) The menu for changing the mode is located at the upper left corner in Blender. F) The selected nanoprocesses are displayed as point clouds (black points) or, if selecting from a polygonal surface mesh, as meshes (gray structures, not shown).

2.2 Transformer

This tool allows transforming a surface point cloud into a polygonal surface mesh and vice versa. Depending on the application, it may be useful to convert the morphology from a point cloud to a mesh to run simulations, or calculate different morphometric properties, for example. Various applications require the morphology to be presented as an equidistant point cloud. The tool for point cloud generation allows for creating an equidistant point cloud onto the mesh surface. The density of the point tiers along any of the three axes in the three dimensional space can be adjusted. The Blender particle system grid is utilized to achieve the equidistant point cloud. The points are arranged symmetrically and their density is defined by the largest dimension of the mesh (either the x, y, or z axis). By default, the Blender grid particle system allows only the maximum resolution of 250; in addition, the density cannot be adjusted to any direction. This tool for point cloud generation implements segmentation of the mesh to produce point clouds that can exceed the resolution limit 250, as well as be made sparser along any of the three axes.

The higher resolution of the point cloud is achieved by creating a frame for the object and dividing the frame (as well as the object within it) into smaller cubical segments. The frame serves to define the resolution of the point cloud but does not affect the alignment of the points. Point clouds are created separately for these smaller segments and finally combined together into one unified point cloud for the object. (Please, note that as the final point cloud is a combination of an even number of smaller point clouds, the final resolution is always an even number. If the user inputs an odd number as the resolution, it will be rounded up to the closest even number.) The point cloud is sparsified along any of the three coordinate axes by simply removing tiers of points.

To transform a surface point cloud to a polygonal surface mesh, open the "Transformer" subpanel and click "Point Cloud to Mesh" (Figure 3A). Click the folder icon to select a point cloud (.dat) and click "Import". For the mesh to be reconstructed correctly, the shape should consist of loops of points. Figure 3B showcases a nanoprocess selected with the first tool from a point cloud taken from Savtchenko et al. (2018). Click "Create Mesh" to convert the nanoprocess from a point cloud to a mesh. The mesh surface can be subdivided with Catmull-Clark algorithm by pressing "Mesh Modifications" to open a subpanel with options for setting the level of subdivision (click "Subdivide" to perform subdivision and "Apply" to apply it). By clicking "Save Mesh" the mesh is saved into a subdirectory within the input directory. The obtained mesh is used below for creating a new, denser point cloud for the same nanoprocess (Figure 3D). The surface meshes created from the point cloud consist of triangular polygons.

To transform a polygonal surface mesh to a point cloud, open the "Transformer" subpanel and click "Mesh to Point Cloud" (Figure 3C). Specify the parameters for the point cloud to be generated:

1. Click the folder icon to select a polygonal surface mesh (.obj) and click "Import". The shape can be rotated by pressing the key "R", moving the mouse, and clicking the left mouse button. To specify rotation along an axis, click the key "R" and then "X", "Y", or "Z".
2. Press "Mesh modification" to open a subpanel with options for remeshing (you can specify the octree depth and scale of remeshing, visualize the result by clicking "Remesh", and apply it by clicking "Apply") and removing segments that are disconnected from the main shape (option "Clean Mesh"). In the case where the mesh consists of exceedingly many small faces, remeshing may help ensure that no incorrectly placed points are created. The two parameters for remeshing, the octree depth and scale, define the resolution of the mesh; for both parameters higher values reproduce the mesh in finer detail.
3. Enter grid resolution (must be an even number).

4. Keep every nth point cloud tier in X/Y/Z direction. To sparsify the point cloud, enter a larger number than 1.

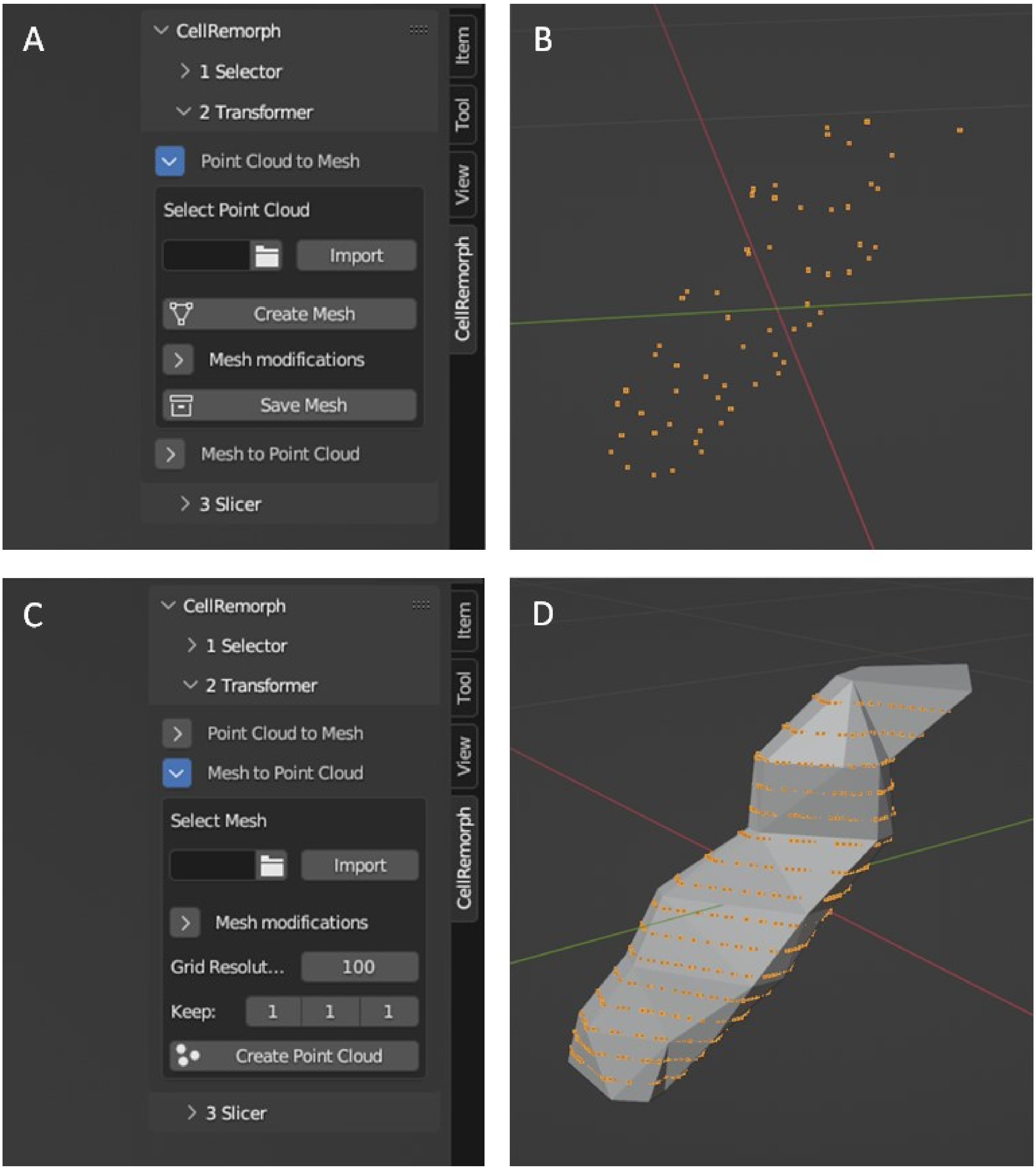

Figure 3. A) The "Transformer" subpanel and the option "Point Cloud to Mesh" opened. B) A nanoprocess selected from a point cloud taken from Savtchenko et al. (2018). C) The "Transformer" subpanel and the option "Mesh to Point Cloud" opened. D) A polygonal surface mesh created from the point cloud in B and a new, denser point cloud created based on the mesh with the following point cloud parameters: grid resolution 100 and sparsify 1, 1, and 6.

Click "Create Point Cloud" to execute. The results are showcased in the Blender main view and saved into subdirectories within your input directory in 3D object format (.ply) and the point locations in text format (.dat). A PLY-formatted point cloud can be imported into Blender by clicking File/Import/Stanford (.ply) and selecting the file. The parameters used and the elapsed times are saved into an output-file (.dat). Example

result of the tool is presented in Figure 3D. The Blender outliner context menu (in the upper right corner) contains the object and the created point cloud within a subcollection under the “Scene Collection”. To rotate or move the object and the point cloud together, press the key “A” first.

2.3 Slicer

Polygonal surface mesh structures can be sliced into desired numbers of segments equaling either in surface area or volume. The segmentations can be executed along any of the three axes. Slicing by surface area or volume functions otherwise according to the same principles, except that slicing by volume adds surfaces onto the sliced structures while slicing by surface area leaves them hollow. The resulting segments are useful, for example, for studying the effect of cell morphology on biophysical dynamics by utilizing the cell segments of equal volumes or surface areas in the simulations.

Open the “Slicer” subpanel (Figure 4A).

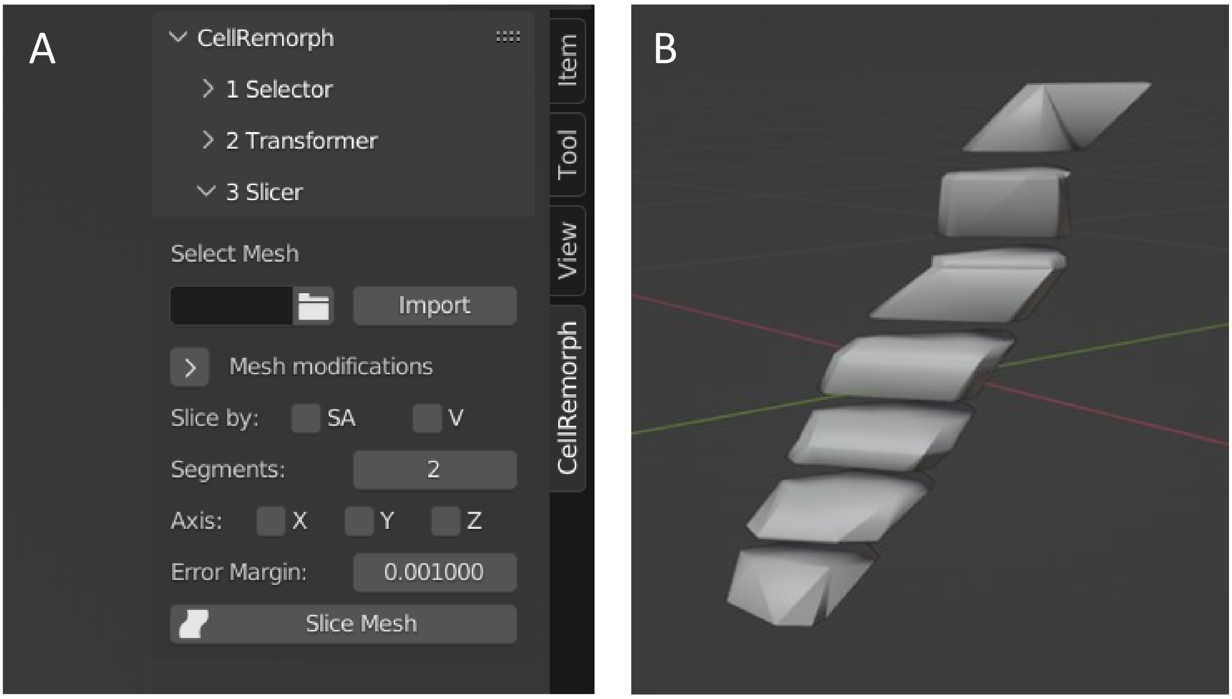

Figure 4. A) The “Slicer” subpanel opened. B) Example result of slicing by volume with the parameters z-axis, 7 segments, and error margin 0.00001. The segments were separated for clearer visualization. The morphology used here is a nanoprocess selected and converted into a polygonal surface mesh from a point cloud taken from Savtchenko et al. (2018).

Specify the parameters for the slicing:

1. Click the folder icon to select a polygonal surface mesh (.obj) and click “Import”. The shape can be rotated by pressing the key “R”, moving the mouse, and clicking the left mouse button. To specify rotation along an axis, click the key “R” and then “X”, “Y”, or “Z”.
2. Press “Mesh modification” to open a subpanel with options for remeshing (you can specify the octree depth and scale of remeshing, visualize the result by clicking “Remesh”, and apply it by clicking “Apply”) and removing segments that are disconnected from the main shape (option “Clean

Mesh”). The two parameters for remeshing, the octree depth and scale, define the resolution of the mesh; for both parameters higher values reproduce the mesh in finer detail.

3. Choose to slice either by surface area (SA) or volume (V).
4. Enter the desired number of segments.
5. Select the axis by which the mesh will be divided (select only one!)
6. Specify error margin.

Click “Slice Mesh” to execute. The results are showcased in the Blender main view and saved into subdirectories within your input directory in 3D object format (.obj). The parameters used and the elapsed times are saved into an output-file (.dat). Example result of the tool is presented in Figure 4B. The segments can be moved by clicking them with the left mouse button, pressing “G”, moving the mouse, and released by clicking with the left mouse button again. The Blender outliner context menu (in the upper right corner) contains the morphology segments within a subcollection under the “Scene Collection”. To rotate or move all of the segments together, press the key “A” first. Alternatively, multiple segments can be selected from the outliner context menu by pressing “Ctrl” while clicking the segment name.

3. How the surface point clouds are transformed into meshes

First the algorithm transforms each planar set of points in the surface point cloud (Figure 5A) into convex hulls (Figure 5B). The convex hull of each planar set of points is the minimum convex set enclosing them all, producing a polygon connecting the outermost points in the plane. In order to get an edge loop containing only the outermost edges, the other edges within the convex hull must be deleted. That is achieved by first finding and deleting the intersecting edges.

The intersections between edges are found by checking the intersection between each possible pairing of edges. The intersection is found for each two lines AB and DC with the help of the algorithm found in https://bryceboe.com/2006/10/23/line-segment-intersection-algorithm/.

First, the function ccw(A,B,C) is defined below and it returns true if vertices A, B, and C are in a counterclockwise order:

ccw(A,B,C) = (C[1]-A[1]) * (B[0]-A[0]) > (B[1]-A[1]) * (C[0]-A[0]).

Second, the expression below returns true if there is an intersection between the two lines AB and CD:

ccw(A,C,D) != ccw(B,C,D) and ccw(A,B,C) != ccw(A,B,D).

After the intersecting edges are deleted, the convex hull is searched for any vertices that give rise to three or more edges. After finding those vertices, the redundant edges (those that are connected to two vertices that give rise to three or more edges) are deleted. The new edge loops consist only of the outermost edges (Figure 5C).

Finally, the edge loops are joined together, and the mesh is triangulated to produce the final surface mesh for the nanoprocess (Figure 5D).

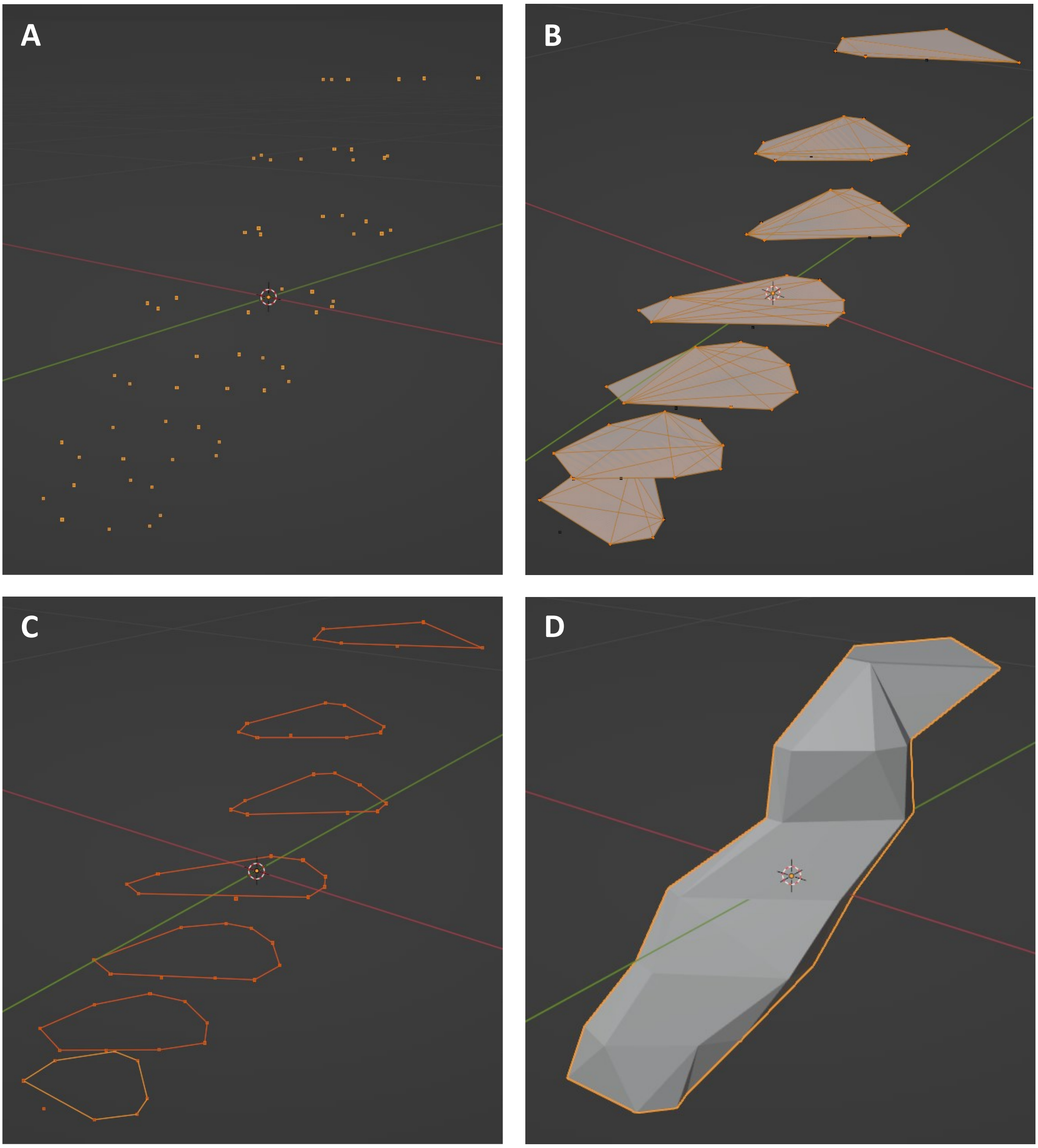

Figure 5. A) Example surface point cloud of a nanoprocess, consisting of seven planar set of points. B) Each planar set of points converted into convex hulls. C) Each planar set of points converted into edge loops. D) The edge loops joined together into the final mesh.

4. References

Abdellah, M., Hernando, J., Eilemann, S., Lapere, S., Antille, N., Markram, H., & Schürmann, F. (2018). NeuroMorphoVis: A collaborative framework for analysis and visualization of neuronal morphology skeletons reconstructed from microscopy stacks. *Bioinformatics*, *34*(13), i574–i582. https://doi.org/10.1093/bioinformatics/bty231

Ascoli, G. A., Donohue, D. E., & Halavi, M. (2007). NeuroMorpho.Org: A Central Resource for Neuronal Morphologies. *Journal of Neuroscience*, *27*(35), 9247–9251. https://doi.org/10.1523/JNEUROSCI.2055-07.2007

Savtchenko, L. P., Bard, L., Jensen, T. P., Reynolds, J. P., Kraev, I., Medvedev, N., Stewart, M. G., Henneberger, C., & Rusakov, D. A. (2018). Disentangling astroglial physiology with a realistic cell model in silico. *Nature Communications*, *9*(3554). https://doi.org/10.1038/s41467-018-05896-w
